# Plasticity of histamine H_3 _receptor expression and binding in the vestibular nuclei after labyrinthectomy in rat

**DOI:** 10.1186/1471-2202-5-32

**Published:** 2004-09-10

**Authors:** Adrian F Lozada, Antti A Aarnisalo, Kaj Karlstedt, Holger Stark, Pertti Panula

**Affiliations:** 1 Department of Biology, Åbo Akademi University, Biocity, Artillerigatan 6A, FIN-20520 Turku, Finland; 2 Department of ORL, HUCH, Helsinki, Finland; 3 Johann Wolfgang Goethe-Universität Frankfurt am Main, Institut für Pharmazeutische Chemie, Biozentrum, 60439 Frankfurt am Main, Germany; 4 Institute of Biomedicine/Anatomy, POB 63, FIN-00014 University of Helsinki, Finland

## Abstract

**Background:**

In rat, deafferentation of one labyrinth (unilateral labyrinthectomy) results in a characteristic syndrome of ocular and motor postural disorders (e.g., barrel rotation, circling behavior, and spontaneous nystagmus). Behavioral recovery (e.g., diminished symptoms), encompassing 1 week after unilateral labyrinthectomy, has been termed vestibular compensation. Evidence suggesting that the histamine H_3 _receptor plays a key role in vestibular compensation comes from studies indicating that betahistine, a histamine-like drug that acts as both a partial histamine H_1 _receptor agonist and an H_3 _receptor antagonist, can accelerate the process of vestibular compensation.

**Results:**

Expression levels for histamine H_3 _receptor (total) as well as three isoforms which display variable lengths of the third intracellular loop of the receptor were analyzed using *in situ *hybridization on brain sections containing the rat medial vestibular nucleus after unilateral labyrinthectomy. We compared these expression levels to H_3 _receptor binding densities.

Total H_3 _receptor mRNA levels (detected by oligo probe H_3X_) as well as mRNA levels of the three receptor isoforms studied (detected by oligo probes H_3A_, H_3B_, and H_3C_) showed a pattern of increase, which was bilaterally significant at 24 h post-lesion for both H_3X _and H_3C_, followed by significant bilateral decreases in medial vestibular nuclei occurring 48 h (H_3X _and H_3B_) and 1 week post-lesion (H_3A_, H_3B_, and H_3C_). Expression levels of H_3B _was an exception to the forementioned pattern with significant decreases already detected at 24 h post-lesion. Coinciding with the decreasing trends in H_3 _receptor mRNA levels was an observed increase in H_3 _receptor binding densities occurring in the ipsilateral medial vestibular nuclei 48 h post-lesion.

**Conclusion:**

Progressive recovery of the resting discharge of the deafferentated medial vestibular nuclei neurons results in functional restoration of the static postural and occulomotor deficits, usually occurring within a time frame of 48 hours in rats. Our data suggests that the H_3_ receptor may be an essential part of pre-synaptic mechanisms required for reestablishing resting activities 48 h after unilateral labyrinthectomy.

## Background

In rat, deafferentation of one labyrinth (unilateral labyrinthectomy) results in a characteristic syndrome of ocular motor and postural disorders. These disorders have been divided into two categories : One category of symptoms, called static, includes head rotation in both the frontal and horizontal planes and ocular nystagmus [1]. The other category, called dynamic, corresponds to a decreased gain of the vestibulo-ocular and vestibulo-spinal refelxes [1]. Behavioral recovery (e.g., diminished symptoms), encompassing 1 week after unilateral labyrinthectomy, has been termed vestibular compensation [2]. Moreover, the time course of recovery is very different for static and dynamic reflexes: static deficits disappear in one week but dynamic deficits tend to take several months to normalize. Because unilateral labyrinthectomy results in a permanent loss of vestibular inputs from the lesioned side, the compensatory process is assumed to be attributable to the reorganization of the neural network in the central vestibular system [3,4]. Many brain regions including, the medial vestibular nucleus (MVe), are implicated in this process of recovery [5-7].

The focus of our study is the histamine H_3 _receptor that was initially characterized as an autoreceptor controlling histamine synthesis and release [8,9]. Subsequently, as a heteroreceptor, the H_3 _receptor was found to mediate presynaptic inhibition of release of histamine, noradrenaline, serotonin, dopamine, glutamate, GABA and tachykinins [10-13], presumably by inhibiting calcium channels [14-16]. The histamine H_3 _receptor was recently cloned from human [17], monkey [18], rat [19], mouse [20], and guinea pig [21]. Moreover, the receptor was found to have several isoforms [21-26] with differential coupling to second messenger systems and a variation in their distribution in a region-specific manner. The isoforms are formed by alternative splicing of the messenger RNA (mRNA; [22,24]). This study involves analysis of trends observed in mRNA expression levels for the H_3 _receptor (H_3X_, the oligonucleotide probe detecting all H_3 _receptor mRNAs characterized so far) as well as three of the known functionally active isoforms (H_3A_, H_3B_, and H_3C_), described by Drutel et al. [24], during the process of post-lesional plasticity in the central nervous system (CNS).

The primary source of histamine (e.g., ligand for H_3 _receptors) are histaminergic perikarya located exclusively in the tuberomammillary nuclei of the posterior hypothalamus [27,28]; these same neurons send axonal projections to many areas of the brain including the vestibular nuclear complex in rat [29-32]. The fact that the rat vestibular nuclear complex is endowed with the H_3 _receptor was established by use of ligand binding [33,34] and in situ hybridization methods [34].

Evidence suggesting that the H_3 _receptor plays a key role in vestibular compensation comes from studies indicating that betahistine, a histamine-like drug that acts as both a partial histamine H_1 _receptor agonist and an H_3 _receptor antagonist [14,35], accelerates the process of vestibular compensation [32,36]. Furthermore, studies have shown that betahistine treatment results in a reduction of [3H]*N*-α-methylhistamine labelling in the vestibular nuclear complex [37]; these findings suggest that betahistine increases histamine turnover and release by blocking presynaptic H_3 _receptors and inducing H_3 _receptor downregulation [37]. It is noteworthy that dynamic vestibular functions can be modulated by H_3 _receptor ligands, e.g., thioperamide [38]; moreover, thioperamide can affect tonic vestibular functions as well with its demonstrated ability to attenuate barrel rotation in rats following unilateral labyrinthectomy [39]. The forementioned studies make tenable the view that further elucidation of H_3 _receptor regulation is required to fully understand the process of vestibular compensation.

The detailed aim of this study was to characterize the patterning of mRNA expression levels for all possible mRNA splice variants of the H_3 _receptor (H_3X_; as described in [24]) and its isoforms which display different variations of the third intracellular loop (H_3A_, H_3B_, and H_3C_; as described in [24]) in the medial vestibular nucleus (MVe) during the process of vestibular compensation.

Moreover, in this study, we compared the aforementioned trend in mRNA expression levels with H_3 _receptor binding densities to reveal the possible plastic changes in the H_3 _receptor which is responsible for significant constitutive activity also *in vivo *[40], histamine-mediated regulation of neurotransmitter release, and therapeutic effects of betahistine.

## Results

### Expression patterns of total H_3 _receptor and H_3 _receptor isoforms (H_3A_, H_3B_, and H_3C_)

**Figure 1:**
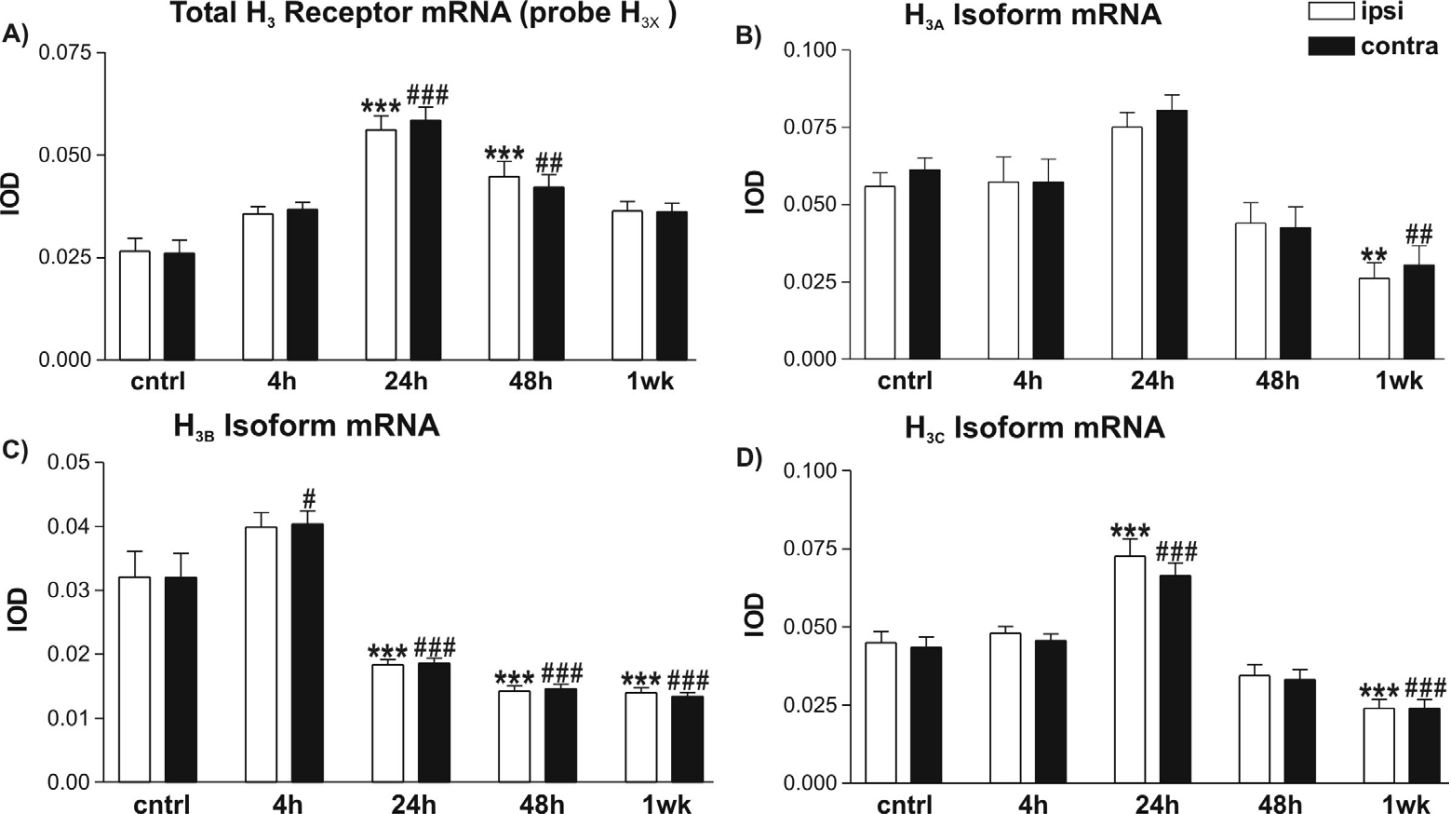
**A, B, C, and D – Expression levels of the total histamine H_3 _receptor (using probe H_3X_) and its three isoforms (H_3A_, H_3B_, and H_3C_) at 4 h, 24 h, 48 h, and 1 week post-lesion. **Data are presented as mean IOD ± SEM. Abbreviations are as follows: ipsi, ipsilateral; contra, contralateral; cntrl, control (n = 4); 4 h, animals sacrificed 4 h post-lesion (n = 4); 48 h, animals sacrificed 48 h post-lesion (n = 4); and 1 wk, animals sacrificed 1 week post-lesion (n = 5). ***p < 0.001 and **p < 0.01 when compared to ipsilateral control. ###p < 0.001, ##p < 0.01, and #p < 0.05 when compared to contralateral control.

Changes in mRNA expression levels for H_3 _receptor and the three H_3 _receptor isoforms (Figure 1A,1B,1C and 1D, respectively) occured bilaterally; that is, there were no significant differences detected between the ipsilateral and contralateral medial vestibular nuclei of animals in all groups studied (e.g., control, 4 h post-lesion, 24 h post-lesion, 48 h post-lesion [n = 4, for each group], and 1 week post-lesion [n = 5]).

**Figure 2:**
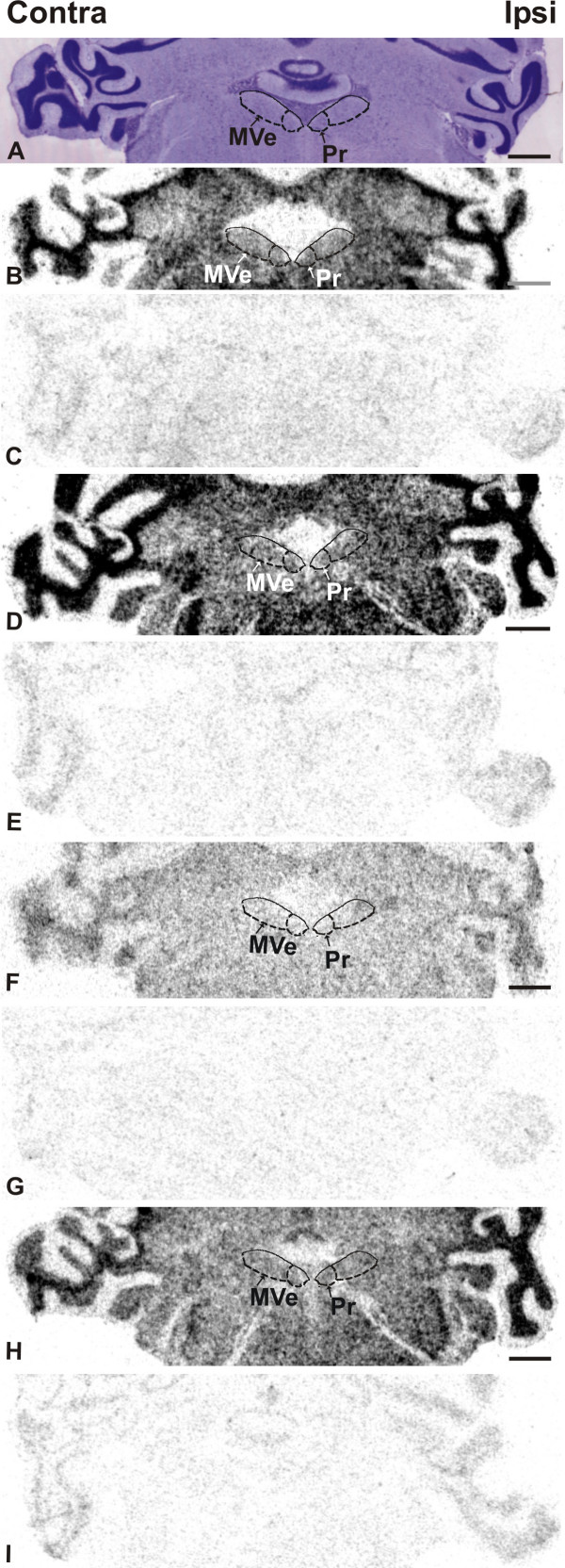
**A) Location of the MVe as visualized on cresyl violet stained section and on x-ray images of parallel sections used in *in situ *hybridization experiments with the various oligonucleotide probes: B) H_3X _C) H_3X _blocking control D) H_3A _E) H_3A _blocking control F) H_3B _G) H_3B _blocking control H) H_3C _I) H_3C _blocking control. **Sections are from a representative animal sacrificed 24 h post-lesion. Abbreviations are as follows: MVe, medial vestibular nucleus and Pr, prepositus nucleus. Scale bars are 100 μm.

We compared the total H_3 _receptor mRNA expression levels (using probe H_3X_) in ipsilateral MVe of control animals to that of test animals from the four time points (Fig. 1A). Fig. 2B shows scanned X-ray film depiction of H_3X _expression in a representative animal 24 h post-lesion. We found no significant rise in H_3X _mRNA levels in the ipsilateral MVe 4 hours after lesioning. Significant increases in H_3X _mRNA levels were found to occur at 24 and 48 hours post-lesion. After 1 week post-lesion, H_3 _receptor mRNA expression levels (as indicated by probe H_3X_) returned to normal levels. The trend observed was similar when comparing H_3X _mRNA levels in contralateral MVe of control animals with that of test animals from the four time points. There was no significant change in total H_3 _receptor mRNA levels detected in contralateral MVe 4 hours post-lesion, but we found a significant increase in H_3X _mRNA levels detected in contralateral MVe both 24 hours and 48 hours post-lesion. Finally, a return to normal levels in total H_3X _receptor mRNA level was detected in contralateral MVe 1 week post-lesion.

No significant increases in H_3A _mRNA expression levels were detected when we compared ipsilateral MVe in control animals with ipsilateral MVe 4 hours, 24 hours, and 48 hours post-lesion (Fig. 1B). Fig. 2D shows scanned X-ray film depiction of H_3A _expression in a representative animal 24 h post-lesion. On the other hand, a significant decrease of H_3A _mRNA levels does occur in the ipsilateral MVe 1 week after lesioning. The trend was identical when comparing H_3A _mRNA levels in contralateral MVe of control animals with H_3A _mRNA levels in contralateral MVe of animals from the four time points: There was no significant increase in H_3A _mRNA levels when comparing to contralateral MVe 4 hours, 24 hours, and 48 hours post-lesion; on the other hand, there is a significant decrease in H_3A _mRNA levels when comparing to contralateral MVe 1 week after lesioning.

No significant changes in H_3B _mRNA expression levels were detected when we compared ipsilateral MVe in control animals to ipsilateral MVe 4 hours post-lesion (Fig. 1C). Fig. 2F shows scanned X-ray film depiction of H_3B _expression in a representative animal 24 h post-lesion. Significant decreases in mRNA levels were found in the ipsilateral MVe 24 hours, 48 hours, and 1 week after lesioning. When comparing contralateral MVe of control animals to contralateral MVe of animals in the other time points, there was a significant increase detected 4 hours post-lesion and this was followed by significant decreases detected at 24 hours, 48 hours after lesion, and 1 week post-lesion.

No significant changes in H_3C _mRNA levels were detected when we compared ipsilateral MVe in control animals to ipsilateral MVe 4 hours post-lesion (Fig. 1D). Fig. 2H shows scanned X-ray film depiction of H_3C _expression in a representative animal 24 h post-lesion. A significant increase was detected when comparing ipsilateral MVe of control animals to ipsilateral MVe 24 hours post-lesion; moreover, the decrease in H_3C _mRNA levels in the ipsilateral MVe 48 hours post-lesion was not significantly different from those of the ipsilateral MVe in control animals. Finally, in comparison to the ipsilateral MVe in controls, there was a decrease in mRNA levels that was found to be significant in the ipsilateral MVe 1 week post-lesion. Results were similar when comparing the contralateral MVe in control animals to contralateral MVe from the other time points: There was no significant increase in H_3C _mRNA levels when comparing to contralateral MVe 4 hours post-lesion, there was a significant increase detected when comparing to contralateral MVe 24 hours post-lesion, the decrease was not significant comparing to contralateral MVe 48 hours post-lesion, and a significant decrease was detected when comparing to contralateral MVe 1 week post-lesion.

### [^125^I]iodoproxyfan Binding Densities

**Figure 3:**
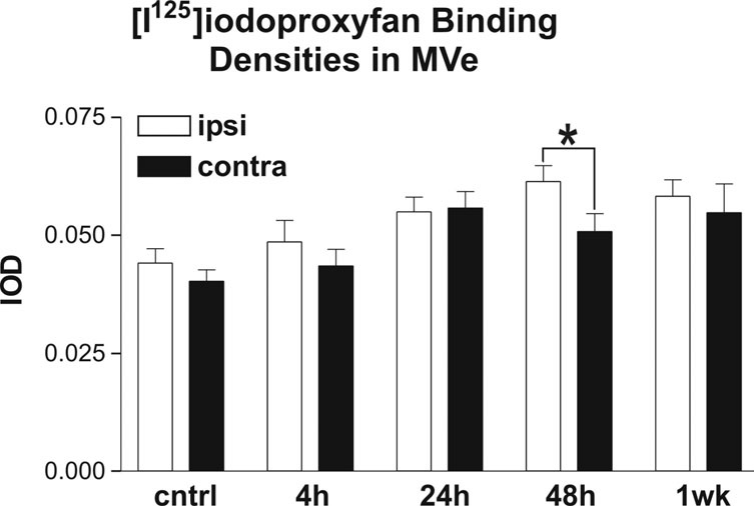
**[^125^I]iodoproxyfan binding densities in MVe of labyrinthectomized rats. **Data are presented as mean IOD ± SEM. Abbreviations are as stated in Figure 1 legend. Sample sizes are as follows: control, n = 3; 4 h post-lesion, n = 4; 24 h post-lesion, n = 4; 48 h post-lesion, n = 4; 1 week post-lesion, n = 5. *p = 0.0193.

**Figure 4:**
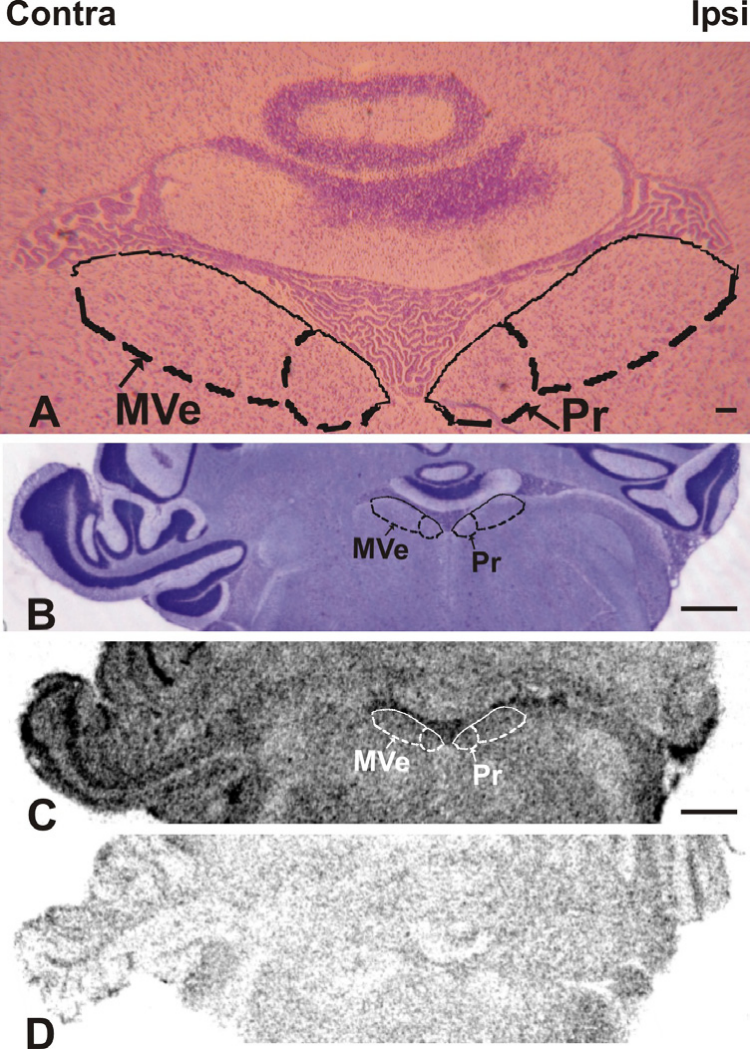
**A) Location of the MVe as visualized at high magnification and B) at magnification used in analyses on cresyl violet stained section and C) on x-ray images of parallel sections used in [^125^I]iodoproxyfan binding experiments; D) non-specific binding control is represented in this image. **Sections are from a representative animal sacrificed 48 h post-lesion. Abbreviations are as stated in Figure 2 legend. Scale bars are 100 μm.

No significant changes were found in H_3 _receptor binding densities (Fig. 3) between ipsilateral MVe in control animals (n = 3) and ipsilateral MVe of animals of different time points. The results were identical when H_3 _receptor binding densities in contralateral MVe in control animals were compared to that of contralateral MVe at different times post-lesion. On the other hand, when comparing the ipsilateral to contralateral MVe 48 hours post-lesion, a significant increase in binding densities occurred on the ipsilateral side (Fig. 4 shows an example of H_3 _receptor binding densities in a representative animal 48 h post-lesion).

## Discussion

Knowing that there is no reliable method to determine the efficiency of a probe for its targetted sequence in an mRNA of interest, this study focuses instead on the pattern of expression for the total H_3 _receptor (detected by using probe H_3X_) and its isoforms (H_3A_, H_3B_, and H_3C_) occurring during the process of post-lesional plasticity in the CNS. Moreover, the probes used in this study were designed to detect unique areas in the transcripts (H_3A_), or junctional areas in deletion isoforms (probes H_3B _and H_3C_) which would make it highly unlikely for non-specific hybridization would occur. This study also includes a comparison of the aforementioned patterns of expression with binding densities for the H_3 _receptor.

The prepositus nuclei are delineated and mentioned in legends for figures 2 and 4. These nuclei are delineated for the sole purpose of giving the reader an idea of the dorsoventral extent of the MVe at the levels depicted in figures 2 and 4. Noteworthy, is that these nuclei are not included in the main functional projections in the vestibulo-ocular and vestibulo-spinal pathways from the brainstem vestibular nucleus in mammals reviewed by Smith and Curthoys [2].

There are three previously described types of neurons in the medial vestibular nucleus [41,42]; in vitro, all three types are depolarized by histamine [43,44]. Moreover, this depolarization has been shown to be exclusively mediated through postsynaptic H_2 _receptors [43,44] suggesting a presynaptic localization of H_3 _receptors in the medial vestibular nuclei [38]. Consequently, there are three possible locations for H_3 _receptors in the MVe: 1) On the histaminergic or other incoming fibers innervating the MVe [29-32]. 2) On terminals of the inhibitory interneurons in the MVe that make synaptic contacts on second order excitatory neurons-defined as neurons in the vestibular nuclei that receive inputs from sensory afferents [45]. 3) On the terminals of second order excitatory MVe neurons making cross-commissural synaptic contacts on contralateral MVe inhibitory interneurons [45]. With respect to MVe on the lesioned side, an H_3 _receptor mediated inhibition of GABA release from inhibitory interneurons or glutamate release from terminals of second order excitatory MVe neurons may underlie the restoration of resting activity in the deafferentated MVe; more precisely, H_3 _receptor action would result in disinhibition of neurons in the deafferentated MVe

With respect to the first possible location, it has been established that histamine fibers are endowed with H_3 _receptors that function as autoreceptors and inhibit histamine release [11,13]. Support for the notion that H_3 _receptors are at the next two possible locations (i.e., either at the terminals of the inhibitory interneurons or at the terminals of second order MVe neurons) comes from work showing that betahistine antagonizes the excitatory effect of histamine on vestibular neurons from *in vitro *slice preparations of the dorsal brainstem of the rat [46]. This finding is significant given that H_3 _receptors mediate presynaptic inhibition of release of other neurotransmitters including: noradrenaline, serotonin, dopamine, glutamate, GABA and tachykinins [10-13]. Unilateral labyrinthectomy induced changes in expression levels of receptors for glutamate (e.g., NR1 and NR2A-D subunits of the NMDA receptor [47] and GluR2-4 subunits of the AMPA receptor [48]) have been studied in the vestibular nuclei. Moreover, the existence of both GABA_A _and GABA_B _receptors in the vestibular nuclei and their involvement in vestibular compensation has been demonstrated by either unilateral perfusion or microinjection of GABAergic agonists and antagonists (e.g., GABA, muscimol, and bicuculline) [49].

Consequently, an H_3 _receptor antagonist such as betahistine could either facilitate GABA release from inhibitory interneurons located in the MVe that make synaptic contacts with second order neurons or facilitate glutamate release from terminals of second order MVe neurons that synapse on inhibitory interneurons in the contralateral MVe [31,45]. Either scenario would lead to an inhibition of second order neurons in the MVe and this would explain the observation that betahistine antagonizes the excitatory effect of histamine on vestibular neurons [46].

On the other hand, betahistine administration is reported to induce recovery with a time benefit of 2 weeks relative to control animals after unilateral vestibular neurectomy [32,36]; this is thought to be due to a bilateral increase in histamine release in the MVe [32,37]. The increased histamine would be bound by H_2 _receptors on the perikarya of MVe neurons ipsilateral and contralateral to the lesion resulting in a bilateral increase in activity. This should facilitate behavioral recovery as it is thought that an imbalance in discharge of MVe neurons (30–40 spikes/s in normal animals [4]) ipsilateral and contralateral to the lesion underlies the static postural and occulomotor deficits triggered by unilateral labyrinthectomy [45]. Yet, as stated before, the actions of betahistine would also extend to H_3 _receptors located on glutamatergic terminals of contralateral MVe neurons or on GABAergic terminals of inhibitory MVe interneurons. Antagonism of H_3 _receptors at either site may also act to speed recovery by increasing the amount of GABA output from terminals of inhibitory interneurons and, as a consequence, equalizing neuronal discharge activity of ipsilateral and contralateral MVe.

The trends toward bilateral increases (24 h post-lesion) followed by decreases (48 h post-lesion) in total H_3 _receptor and H_3A_, and H_3C _isoform mRNA levels, with H_3B _mRNA levels already increasing at 4 hours post-lesion and decreasing at 24 h post-lesion, coinciding with a significant increase in H_3 _receptor binding densities in the ipsilateral MVe detected 48 hours post-lesion suggests the occurrence of one or a combination of events in the ipsilateral MVe: 1) an increase in translation has occurred. 2) A change in receptor trafficking between intracellular stores and cell membrane has occurred so that it can be detected as an increase in H_3 _receptor receptor binding densities. In either case, an increase in functional H_3 _receptor protein coupled with an increase in receptor activity may lead to a restoration of resting activity in the deafferentated MVe by scenarios already mentioned in this section.

## Conclusions

Our findings are significant given that normalization of resting activities in neurons located in the ipsilateral MVe has been shown to occur 48 hours after unilateral labyrinthectomy [50]. Moreover, by 48 hours post-lesion, commissural disinhibition has been observed to occur [51]. Placement of H_3 _receptors at the terminals of either GABAergic inhibitory MVe interneurons or on terminals of glutamatergic second order MVe neurons with contralateral projections should result in the observed commissural disinhibition as increased H_3 _receptor activity would result in an inhibition of synaptic release of neurotransmitters.

Our data would thus suggest that H_3 _receptors are involved in presynaptic mechanisms resulting in a normalization of resting activities in ipsilateral MVe neurons which would balance discharge activity in MVe on both sides.

## Methods

### Animals and surgery

This study was approved by the Local Committee for Animal Experiments and the Provincial State Office of Western Finland; in addition, animal experiments were in accordance with the European Convention (1986) guidelines and approved by the Animal Ethics Committee of Abo Akademi Unviversity. Adult male Sprague Dawley rats (200–250 g) were used. Intraperitoneal (ip) injection of pentobarbital (45 mg/kg ip; Mebunat, Orion, Finland) was used as an anesthetic; in addition, local anesthetic Lidocain (Orion, Finland) was infiltrated under the skin and periosteum prior the procedure. Three steps initiated the surgical procedure to left side of the animal's head: retroauricular skin incision, opening of the middle ear bulla with a drill, and removal of the ossicular chain with the aid of a microscope. Unilateral labyrinthectomy was carried out by opening the horizontal semicircular canal duct in the temporal bone, drilling through the horizontal and anterior semicircular canal ampullae and aspirating the contents of the vestibule. 100% ethanol was injected into the opened labyrinth to finalize the procedure; finally, the wound was sutured. The sham operation entailed opening the middle ear bulla and leaving the ossicular chain intact prior to suturing the wound, as described in Cameron and Dutia [52]; the control animals (n = 4) that underwent the sham operation were killed 4 h later. The method is explained hereafter. All rats included in the experiments displayed symptoms characteristic of animals that have undergone unilateral labyrinthectomy (e.g., barrel rotation, circling behavior, and spontaneuos nystagmus). These symptoms gadually disappeared during the first two or three days and were completely absent within one week. Animals were stunned by CO_2 _gas and killed by decapitation 4 h (n = 4), 24 h (n = 4), 48 h (n = 4) and 1 week (n = 5) after labyrinthectomy.

### Tissue preparation

After the mentioned decapitation, brains were removed, frozen in isopentane (-25°C), and stored at -70°C. Tissues were then cut to 20 μm thick cryosections, thaw mounted onto poly-L-lysine coated slides (Menzel-Gläser, Germany), and stored at -70°C until used.

### In-situ hybridization histochemistry

The oligonucleotide probes used for in situ hybridization were designed so that they specifically recognized the different H_3 _receptor isoform mRNAs (H_3A_, H_3B_, and H_3C_; as described in [24]); an additional oligonucleotide probes was used to detect all characterized H_3 _receptor isoforms (H_3X_; as described in [24]). Sequences for H_3X_, H_3A_, H_3B_, and H_3C _probes have been previously published [24]. It is noteworthy that the isoform-specific probes detect selectively the various deletion forms of the third intracellular loop, but do not differentiate between the possible alternative C-termini of the H_3 _receptor isoforms [53]. However, it has been shown that the differences in the third intracellular loop are significant for coupling to intracellular second messengers [24]. As a control, we used a normal hybridization mixture with a 100-fold excess of unlabeled specific probes. As an additional control, we used a *Staphylococcus aureus *chloramphenicol acetyltransferase-specific oligonucleotide probe. The hybridization procedure used has been described before and was used with minor modifications [54,55]. All probes were labeled with [^35^S]deoxyadenosine 5'-α(-thio) triphosphate (New England Nuclear, USA) at their 3' ends using terminal deoxynucleotide transferase (Promega, USA). Nonincorporated nucleotides were removed by purification through Sephadex G-50 columns.

Before hybridization, cryosections were taken from the -70°C environment and kept at room temeprature for 10 min and treated with UV light for 5 min. The hybridization (10^7^cpm/ml) was carried out at 50°C for 16 to 20 hours in a humidified chamber. Posthybridization washes were carried out as described previously [55]. Brain sections from control animals and animals 4 h, 24 h, 48 h, and 1 week post-lesion were treated simultaneously with their respective oligonucleotide probe. Sections and carbon-14 standards were exposed to Kodak BioMax X-ray films (Kodak, USA) for 10 days.

### Receptor binding autoradiography

Autoradiographic localization of [^125^I]iodoproxyfan binding sites has been described before [56]. Briefly, slide mounted tissue sections were preincubated for 15 min in 50 mM Na_2_HPO_4_-KH_2_PO_4 _phosphate buffer, pH 6.8, containing 0.1% bovine serum albumin and 1 μM S132 (a 1-substituted imidazole derivative displaying a very low affinity at H_3 _receptors and used to decrease non-specific labelling). The sections were then incubated for 1 hour at room temperature in the same buffer containing 15 pM [^125^I]iodoproxyfan (Amersham Pharmacia Biotech UK Limited, England). Non-specific binding was determined by incubating consecutive sections in the presence of 1 μM (R)α-methylhistamine (Sigma-Aldrich, Germany). At the completion of incubation, the tissues were washed four times (4 min each) in the same fresh ice-cold buffer, dipped into ice-cold water, and dried under a current of air. Brain sections from control animals and animals 4 h, 24 h, 48 h, and 1 week post-lesion were treated simultaneously. Dried sections, along with standards, were exposed to Kodak BioMax X-ray films (Kodak, USA) for 2 days.

### Image analysis and statistics

Autoradiographic films were quantified by digitizing the film images with a computer based MCID 5+ image analysis system (Imaging Research, Canada) and by measuring gray scale pixel values. The relative optic density was converted to integrated optic density (IOD) based on a standard curve derived from standards exposed to films. Gray scale values were determined from four sections for each animal, measurements from white matter of each respective section were subtracted to obtain the final values, and the data were analyzed using either a paired t-test or one-way ANOVA combined with Bonferroni's Multiple Comparison Test as a post-hoc test. Significance was determined when p < 0.05.

## Authors' contributions

AL assisted with the surgeries and tissue collection, sectioned all of the brains, carried out the *in situ *hybridization and binding studies, performed all of the image and statistical analyses, and participated in the design of the study. AAA performed the surgeries, assisted with tissue collection, assisted with image analyses, and participated in the design of the study. KK established the method of *in situ *hybridization in the lab, designed the oligonucleotide probes, assisted with surgeries and tissue collection, and participated in the design of the study. HS synthesized and supplied rare chemicals required for the binding study. PP acquired funding, coordinated the study, and participated in its design. All authors read and approved the final manuscript.
